# Factors influencing online patient-reported ratings among shoulder surgeons

**DOI:** 10.1016/j.jseint.2025.04.011

**Published:** 2025-05-07

**Authors:** Krishna N. Chopra, Sameer R. Khawaja, Anna L. Gorsky, Nicole L. Greene, Frank L. Vazquez, Joanne Y. Zhou, Zaamin B. Hussain, Michael B. Gottschalk, Eric R. Wagner

**Affiliations:** Department of Orthopaedic Surgery, Emory University School of Medicine, Atlanta, GA, USA

**Keywords:** Shoulder and elbow, Sports medicine, Fellowship, Patient ratings, Online engagement, Academic productivity, Social media, Physician review websites

## Abstract

**Background:**

Patient-reported ratings serve as a model of satisfaction and can play a major role in patient recruitment. Many variables can influence ratings on physician review websites (PRWs), such as social media use, academic productivity, fellowship type, and practice setting. This study examines such factors and their relationship with average ratings and overall patient engagement on PRWs.

**Methods:**

The American Shoulder and Elbow Surgeons directory was queried for all active members who completed either a shoulder and elbow or sports medicine fellowship in the United States. Each name was searched online for professional accounts, and the number of followers was recorded for each. A summated social media presence score was calculated to identify the top 15% of social media users in each cohort. The presence of a practice group or personal website was also recorded, as was a surgeon's practice setting (academic vs. private) and region of practice. H-index was searched on Scopus. Average ratings, number of reviews, and number of comments were collected from Healthgrades, Google Reviews, and Vitals.

**Results:**

A total of 231 shoulder surgeons were included in this review. Compared to shoulder surgeons in academic practice, those in private practice had higher mean ratings and patient engagement on PRWs. When comparing fellowships, shoulder and elbow fellowship–trained surgeons had a greater mean number of ratings on Google than those who completed a sports medicine fellowship. The top 15% of social media users had higher average patient ratings and engagement on Healthgrades compared to the rest of the cohort. There was a positive association between h-index and average rating on Google. No significant associations were found between patient satisfaction and the prestige of medical school or residency program.

**Discussion:**

Several variables can influence patient satisfaction by way of average ratings and engagement on PRWs. Prospective patients are much more likely to select surgeons with higher ratings; thus, shoulder surgeons can use this information to capitalize on opportunities to improve patient retention, recruitment, and overall patient satisfaction.

Patient-reported satisfaction is an essential metric used to represent adequate patient care in medicine. Physicians and hospitals are evaluated and reimbursed based on their ability to meet patient satisfaction standards.^30^ Patient ratings on physician review websites (PRWs) such as Healthgrades, Google Reviews, and Vitals, have become important tools for patients when selecting a practice or physician.^1^^,^^7^ Roughly 60% of patients choose a doctor based on high online ratings, while 61% of patients avoid doctors with poor ratings.^18^^,^^19^ Likewise, since COVID-19, PRWs have established themselves as a fixture for advertising one's practice while retaining current and recruiting new patients.^6^^,^^25^ Online reputations on PRWs play a critical role in how patients choose their providers; thus, it is important for physicians to understand the factors that play a role in patient satisfaction.

Particularly in surgical fields, several factors can impact a patient's experience, satisfaction, and ultimately, their rating.^29^ Positive surgical outcomes and a lack of postoperative complications have been associated with higher patient ratings.^12^^,^^16^ Longer wait times tend to be negatively associated with patient satisfaction,^3^ while empathy and friendly physician–patient communication have been found to be positively associated with patient satisfaction.^4^^,^^26^ Previous studies have found no association between physician gender, ethnicity, and race to patient-reported ratings,^5^^,^^9^ although Lu et al found a negative association between patient satisfaction to several nonmodifiable characteristics.^17^ Nonsurgical factors such as years of experience, practice setting, and prestige of one's medical school and/or residency program, may also play a role in patient satisfaction. A similar study among neurosurgeons found a positive association between Healthgrades ratings and completion of a fellowship, without any association with geographic location, sex, PhD acquisition, or academic status or rank.^9^ Currently, there remains a gap in the literature regarding such factors and their relationship with patient-reported ratings for shoulder surgeons.

This study aims to determine the association between various nonsurgical factors and patient satisfaction among shoulder surgeons who have completed either a sports medicine or shoulder and elbow fellowship. We classify these factors as modifiable, or characteristics that can be controlled by surgeons once they are in practice, to a certain extent (practice type/set-up, research productivity, and social media utilization), and nonmodifiable, or characteristics that cannot be changed by surgeons once they are in practice (training program, level of experience, sex, or region of practice). We hypothesize a positive association between patient satisfaction and factors including experience level, social media usage, research productivity, and the prestige of one's training programs, as all of these can be found online easily by prospective patients and can influence their perceptions of their surgeon. Our secondary hypothesis is that we do not expect an association between patient satisfaction and type of fellowship or region of practice.

## Methods

This study was exempt from Institutional Review Board approval, as it was an observational study based on publicly available information. The American Shoulder and Elbow Surgeon (ASES) directory was queried for all surgeons actively practicing in the United States in 2024. Surgeons outside of the United States, those in active-duty military service, candidate members, or those retired from practice were excluded from this study.

An internet search was conducted using each surgeon's name and degree as recorded in the ASES directory. Demographic information including sex, practice type (private vs. academic), and region of practice (Northeast, Midwest, Southeast, West, and Southwest) was recorded from practice group or personal websites. Private practice was defined as surgeons in a group practice, hybrid academic/private practice, or hospital employment. Academic practice was defined as employment by a university with or without an associated professorship.^13^ Data including medical school, residency program, fellowship type, and the number of years of practice experience since fellowship were recorded for each surgeon using the US News Health Report. The 2021 US News report ranking was consulted to identify the top 20 medical schools, as this was the most recent year the service provided these rankings. The 2024 Doximity reputation rankings were used to identify the top 20 ranked orthopedic surgery residency programs. Each surgeon's h-index was found using Scopus as an estimate of their research productivity.^27^

The methodology used to determine social media utilization is similar to that described by Kerzner et al,^13^ Lander et al,^14^ and Narain et al.^22^ Each surgeon was searched on Google, including their full name, degree, and platform of interest, in adherence of each platform's terms of service. Platforms of interest included public professional social media profiles on LinkedIn, Twitter/X, Instagram, Facebook, TikTok, and YouTube. Institutional YouTube accounts were excluded for standardization, and only those created by surgeons were noted. The presence of a practice group website and a personal website was also noted. Practice group websites are created by the practice and include a surgeon-specific profile, while personal websites are created by the surgeon for education or marketing purposes. A summated online presence score was calculated from 0 to 14, awarding 1 point for having each of the following: practice group website (1), personal website (1), ResearchGate profile (1), LinkedIn profile (1), or public professional profiles on Twitter/X (1), Instagram (1), Facebook (1), TikTok (1), and YouTube (1). An additional point was awarded for each active social media profile (Twitter/X [1], Instagram [1], Facebook [1], TikTok [1], and YouTube [1]). Being active on social media was defined as having posted content on a specific platform within the past 6 months.^22^ Summated online presence scores were used to determine the top 15% of social media users overall to see if social media presence or activity influenced patient satisfaction ratings.

Each surgeon was searched on PRWs including Healthgrades, Google Reviews, and Vitals. Their average ratings, number of ratings, and number of comments on each site was recorded. The presence of a personalized biography on Healthgrades was also noted. The number of comments on Google Reviews was not recorded, as this value is not provided by the website.

Descriptive statistics and frequencies were calculated for all demographic data. Student t-tests were used to compare mean ratings, number of ratings, and number of comments based on the variable of interest. Variables of interest included practice setting, fellowship type, active social media usage, and the prestige of medical school and residency program, among others. Linear regression analysis was used to examine the association between h-index and years of practice to mean ratings, number of ratings, and number of comments on PRWs. Statistical analysis was performed using IBM SPSS (v. 29.0; IBM Corp., Armonk, NY, USA).

## Results

### Surgeon demographics

A total of 231 shoulder surgeons—consisting of 134 shoulder and elbow fellowship–trained and 97 sports medicine fellowship–trained surgeons—were included in this analysis after application of all inclusion and exclusion criteria. Demographic data can be found in Table I.

**Table I:** Shoulder & elbow vs. sports-trained orthopedic surgeon's demographics.

	Shoulder & elbow (n = 134)	Sports medicine (n = 97)	Overall (n = 231)
Sex:- Male, n (%)	129 (96.3%)	94 (96.9%)	223 (96.5%)
Practice type			
Academic, n (%)	58 (43.3%)	50 (51.5%)	108 (46.8%)
Private, n (%)	76 (56.7%)	47 (48.5%)	123 (53.2%)
Years since fellowship, mean ± SD	21.4 ± 9.7	24.8 ± 8.9	22.8 ± 9.5
Region			
Northeast, n (%)	37 (27.6%)	22 (22.7%)	59 (25.5%)
Midwest, n (%)	35 (26.1%)	20 (20.6%)	55 (23.8%)
Southeast, n (%)	34 (25.4%)	27 (27.8%)	61 (26.4%)
West, n (%)	18 (13.4%)	19 (19.6%)	37 (16.0%)
Southwest, n (%)	10 (7.5%)	9 (9.3%)	19 (8.2%)

### Patient satisfaction based on surgeon experience

When looking at experience level, 38.1% of the cohort had greater than 25 years of practice experience (n = 88), 36.4% had 15-24 years of experience (n = 84), and 24.2% had 5-14 years of experience since fellowship (n = 56). There was a negative association between the number of practice years since fellowship and mean ratings on Healthgrades (β = −0.01, *P* = .003; Table VII) and Vitals (β = −0.01, *P* = .008). The number of years since fellowship was negatively associated with the average number of ratings on Google Reviews for each surgeon (β = −1.47, *P* = .03).

**Table II:** Overall ratings and engagement on PRWs based on practice type.

	Private (n = 123)	Academic (n = 108)	*P* value
Healthgrades			
Average ratings	4.4 ± 0.5	4.2 ± 0.8	**.030**
Number of ratings	72.3 ± 84.6	45.0 ± 62.2	**.006**
Number of comments	45.2 ± 57.5	26.7 ± 46.5	**.008**
Google Reviews			
Average ratings	4.7 ± 0.6	4.5 ± 0.8	.072
Number of ratings	86.2 ± 117.2	33.1 ± 53.7	**<.001**
Vitals			
Average ratings	4.3 ± 0.61	4.3 ± 0.6	.470
Number of ratings	50.2 ± 60.5	30.9 ± 32.4	**.003**
Number of comments	26.3 ± 46.0	11.4 ± 13.8	**.001**

*PRWs*, Physician review websites.

Bold *P* value denotes statistical significance.

**Table III:** Overall ratings and engagement on PRWs based on social media usage.

	Top 15% (n = 35)	Bottom 85% (n = 196)	*P* value
Healthgrades			
Average ratings	4.7 ± 0.3	4.6 ± 0.0	**<.001**
Number of ratings	86.0 ± 117.6	54.8 ± 65.3	**.025**
Number of comments	56.1 ± 74.8	33.1 ± 47.9	**.018**
Google Reviews			
Average ratings	4.8 ± 0.3	4.8 ± 0.3	.245
Number of ratings	83.9 ± 104.5	57.4 ± 94.84	.135
Vitals			
Average ratings	4.4 ± 0.5	4.5 ± 0.6	.645
Number of ratings	43.9 ± 60.0	40.7 ± 47.1	.736
Number of comments	21.0 ± 37.6	19.0 ± 35.4	.761

*PRWs*, Physician review websites.

Bold *P* value denotes statistical significance.

**Table IV:** Overall ratings and engagement on PRWs based on fellowship training.

	Shoulder & elbow (n = 134)	Sports medicine (n = 97)	*P* value
Healthgrades			
Average ratings	4.4 ± 0.7	4.3 ± 0.5	.306
Number of ratings	64.7 ± 79.1	52.4 ± 71.4	.228
Number of comments	41.3 ± 56.9	30.1 ± 47.5	.116
Google Reviews			
Average ratings	4.6 ± 0.6	4.5 ± 0.9	.376
Number of ratings	71.7 ± 111.4	47.1 ± 69.4	.055
Vitals			
Average ratings	4.4 ± 0.6	4.3 ± 0.6	.282
Number of ratings	40.3 ± 54.5	42.4 ± 43.8	.755
Number of comments	20.3 ± 41.3	17.9 ± 26.0	.622

*PRWs*, Physician review websites.

**Table V:** Overall ratings and engagement on PRWs based on medical school ranking.

	Attended top 20 school (n = 65)	Did not attend top 20 school (n = 166)	*P* value
Healthgrades			
Average ratings	4.4 ± 0.8	4.3 ± 0.6	.496
Number of ratings	58.8 ± 82.1	59.8 ± 73.8	.926
Number of comments	35.7 ± 56.7	36.9 ± 52.1	.883
Google Reviews			
Average ratings	4.6 ± 0.6	4.6 ± 0.7	.957
Number of ratings	55.3 ± 86.7	63.8 ± 100.4	.551
Vitals			
Average ratings	4.4 ± 0.6	4.3 ± 0.6	.428
Number of ratings	38.7 ± 41.1	42.2 ± 53.4	.640
Number of comments	14.8 ± 21.6	21.1 ± 39.7	.226

*PRWs*, Physician review websites.

**Table VI:** Overall ratings and engagement on PRWs based on residency program ranking.

	Attended top 20 program (n = 89)	Did not attend top 20 program (n = 142)	*P* value
Healthgrades			
Average ratings	4.4 ± 0.6	4.3 ± 0.7	.145
Number of ratings	66.0 ± 83.1	55.5 ± 71.3	.306
Number of comments	40.0 ± 58.7	34.4 ± 49.7	.436
Google Reviews			
Average ratings	4.5 ± 0.8	4.6 ± 0.7	.524
Number of ratings	58.2 ± 75.1	63.4 ± 108.1	.691
Vitals			
Average ratings	4.4 ± 0.6	4.3 ± 0.6	.062
Number of ratings	41.3 ± 40.3	41.2 ± 55.7	.981
Number of comments	17.8 ± 23.4	20.3 ± 41.5	.599

*PRWs*, Physician review websites.

**Table VII:** Multivariate regression analysis of Healthgrades ratings with practice experience and H-index.

	Mean rating		Mean number of ratings		Mean number of comments	
	Estimate (b)	*P* value	Estimate (b)	*P* value	Estimate (b)	*P* value
Years since fellowship	−0.01	**.003**	−0.29	.590	−0.61	.100
H-index	0.00	.914	0.36	.133	0.18	.292

Bold *P* value indicates statistical significance.

### Patient satisfaction based on practice setting

Of the 231 shoulder surgeons, 123 (53.2%) were in private practice and 108 (46.8%) were in academic practice (Table II). Surgeons working in private practice demonstrated higher mean ratings (*P* = .030), number of ratings (*P* = .006), and number of comments (*P* = .008) on Healthgrades compared to academic surgeons. Private practice surgeons also had a higher mean number of ratings compared to academic surgeons (*P* < .001) on Google Reviews. On Vitals, private practice shoulder surgeons had a higher mean number of ratings (*P* = .003) and number of comments (*P* = .001) than academic surgeons.

### Patient satisfaction based on social media usage

There were 35 surgeons who accounted for the top 15% of social media users across the overall cohort. Increased social media usage was associated with higher mean ratings (*P* < .001), a higher number of ratings (*P* = .025), and a higher number of comments (*P* = .018) on Healthgrades (Table III). There were no associations noted between social media usage and satisfaction ratings or engagement on Vitals or Google Reviews. Of the 35 surgeons in the top 15% of social media users, 17 (48.6%) had 5-14 years of practice experience. The next highest represented age group in this cohort was made up of 12 surgeons who had 15-24 years of experience (34.3%).

Surgeons with personalized Healthgrades biographies had higher ratings on average (4.53 ± 0.44) than those with a standard template biography (4.25 ± 0.69; *P* = .003). Those with a personalized biography also had a higher mean number of ratings (96.41 ± 102.08 vs. 44.80 ± 56.70; *P* < .001) and comments (65.08 ± 70.40 vs. 25.14 ± 39.55; *P* < .001) compared to those without a custom biography. Having a personal or practice group website was not associated with higher patient ratings or engagement on PRWs.

### Patient satisfaction based on research productivity

Academic shoulder surgeons had a higher mean h-index than those in private practice, 30.19 vs. 22.58 (*P* = .048), respectively. While h-index was positively associated with average ratings on Google Reviews (β = 0.005, *P* = .03), there were no other significant associations found between h-index and patient satisfaction variables.

### Patient satisfaction based on training prestige

When looking at program prestige, 65 (28.1%) of 231 shoulder surgeons attended a top 20 medical school. In comparing the cohort of surgeons who attended a top 20 institution vs. not, both groups had statistically similar h-indexes, 31.57 ± 21.03 vs. 26.64 ± 20.62 (*P* = .11). 89 (38.5%) shoulder surgeons trained at a top 20 orthopedic surgery residency program, while 142 (61.5%) did not. Surgeons who attended a top 20 residency program had a higher h-index (38.83 ± 24.93) compared to those who did not (23.76 ± 16.46; *P* < .001). Attending a highly ranked medical school and/or residency program was not found to be associated with higher patient satisfaction or engagement across all three PRWs of interest (Tables V and VI).

### Patient satisfaction based on fellowship type

A total of 134 (58.0%) shoulder and elbow fellowship–trained and 97 (42.0%) sports medicine fellowship–trained shoulder surgeons were compared. No differences were found in patient satisfaction based on fellowship type (Table IV).

### Patient satisfaction based on region of practice

The distribution of shoulder surgeons based on region of practice can be found in Table I. No significant associations were discovered between region of practice and patient satisfaction across all three PRWs of interest. A summary of all factors related to patient satisfaction is displayed in Table VIII.

**Table VIII:** Associations of factors that are related to increased patient satisfaction.

Associated		Not associated	
Modifiable	Nonmodifiable	Modifiable	Nonmodifiable
Practice type/set-up	-	H-index	Region of practice
Social media usage			Medical school ranking
Customized biographies on PRWs			Residency program ranking
Years of experience			Fellowship type

*PRWs*, Physician-rating websites.

## Discussion

In an era where information is easily accessible to the public, patient satisfaction ratings on PRWs are as important as ever for physicians.^20^ Reviews left by patients can significantly influence a prospective patient's decision on whether to see a surgeon or practice. Likewise, patient satisfaction remains an important factor in patient retention,^2^ surgeon and hospital reputation,^8^ quality improvement initiatives,^24^ and ultimately, patient outcomes.^10^ The influence of nonsurgical factors that affect patient satisfaction for physicians has yet to be discussed. The purpose of this study was to identify factors that play a role in patient satisfaction for shoulder surgeons.

Factors such as surgeon experience, practice type, social media utilization, and research productivity can affect patient satisfaction.^4^^,^^26^^,^^29^ Interestingly, experience level, determined by number of practice years since fellowship, was negatively associated with patient ratings and engagement on PRWs, suggesting a possible preference for younger surgeons. This is also supported by the fact that in our study, surgeons with 5-14 years of practice experience made up 48.6% of the top 15% of social media users overall, suggesting that younger surgeons have a proclivity toward social media usage. Practice type was also associated with patient ratings and engagement online. Of course, this could be due to the fact that private practices must work actively to recruit patients, so they tend to dedicate more time and effort toward tools such as social media to bring in patients. Our study found that shoulder surgeons in private practice demonstrated significantly higher patient ratings and engagement across PRWs compared to surgeons in academic practice. These findings may potentially reflect differences in how surgeons and practices intentionally create a work and clinic culture prioritizing patient retention and recruitment. One such example is private practice emphasis on practice design, which is the process of basing decisions about the physical environment of the practice on credible research to achieve optimal outcomes.^11^ Attention to office layout through way of public signage, front desk and waiting area design, and music can decrease patient stress, increase compliance with treatment plans, and ultimately boost patient satisfaction, referrals, and retention.^11^ Academic institutions can optimize the patient experience through improving the cosmetic design of advertising, waiting rooms, patient rooms, and more. Investing in the physical design and décor of a practice has been found to significantly benefit office functionality, staff performance, and overall patient satisfaction.^11^ It is also important to consider that academic institutions may place restrictions on their physicians utilizing social media, which could contribute to private practice surgeons being more active on social media and therefore having higher patient satisfaction ratings. Another factor to consider in understanding the relationship between patient satisfaction and practice type is that private practices often employ social media managers or other specialists to promote the practice online and encourage satisfied patients to leave reviews on PRWs, leading to increased patient engagement.

Other factors like social media engagement, personable profiles, and research productivity can also contribute to increased patient satisfaction. In the post–COVID-19 era, social media has established itself as a vital marketing tool for patient recruitment, education, and retention.^25^ The top 15% of social media users had higher patient ratings and engagement on PRWs compared to their colleagues with little to no social media presence. This could be explained by the fact that physicians who emphasize social media activity are more likely to value their online presence and work to encourage patient engagement and enhance satisfaction on PRWs. In addition, surgeons who invest time to write personalized biographies on Healthgrades had higher ratings, number of ratings, and number of comments on the site, indicating a low-commitment–high reward method to improving patient satisfaction online. Something as simple as a personalized biography can lead to more personable connections between patients and their physicians, with patients feeling like they better understand their surgeons. Finally, h-index, which has been established as a method to quantify academic productivity and engagement among researchers,^27^ was positively associated with patient ratings on Google Reviews. This association may suggest a possible preference for surgeons with a research background, based on the idea that surgeons who actively engage in research may be the most up to date on advances in their specific field and rely on evidence-based outcomes to dictate their plan of treatment.

It is also important to note that several factors were found to not be associated with higher patient satisfaction. Fellowship type, prestige of a surgeon's medical school and residency program, and region of practice did not influence patient ratings or engagement on PRWs. While program prestige may be important for individuals to advance to the next level of training (medical school to residency, residency to fellowship, and fellowship to practice), our findings indicate that patients may not take it into consideration when selecting or rating their shoulder surgeon.

Previous literature has focused on several other factors that may play a role on patient satisfaction in orthopedics. Korth et al found the presence of a physician assistant to positively affect patient satisfaction with outpatient orthopedic surgery, likely improving the perception of care.^28^ They did not find the presence of a fellow or nurse practitioner, provider distance from home, or productivity (measured as total relative value units) to play a role in patient satisfaction.^28^ Lee et al found that patients who received flowers from their surgeon following arthroplasty procedures reported higher Press Ganey survey scores, suggesting that symbolic gifts can increase feelings of loyalty toward one's surgeon.^15^ Lu et al determined nonmodifiable orthopedic surgeon characteristics, such as being female, Asian, and unmarried to be associated with reduced Press Ganey scores.^17^ These results represent unconscious biases that may negatively impact patient–physician interactions, physician ratings on PRWs, and ultimately, surgeon reputation. Unlike our findings for shoulder surgeons, they did not find an association between experience level and research productivity to patient satisfaction scores,^17^ though such discrepancies may be due to their limited sample size.

Our findings provide valuable insights on factors that influence patient attitudes toward their shoulder surgeon, impacting patient engagement and recruitment. Social media utilization has slowly grown among orthopedic surgeons,^13^^,^^22^ proving to be a valuable tool for patient education and recruitment. Higher patient ratings for younger shoulder surgeons and those with greater research productivity can indicate a preference for surgeons taking part in the newest innovations in shoulder surgery or for those taking the time to capitalize on social media usage as a patient recruitment tool. Surgeons, regardless of age, can continue to engage with recent literature to provide patients with valuable information. Finally, our findings demonstrate no association between nonmodifiable factors including training program prestige and patient ratings, indicating that patients are unlikely to consider where their surgeon completed their training when reporting satisfaction. This may provide reassurance to those dealing with the increasing competitiveness of medical school and orthopedic surgery residency admissions that program choice will not significantly impact patient satisfaction.^23^

While this study has thoroughly examined the relationship between factors that influence patient satisfaction among shoulder surgeons, there are limitations that must be considered before drawing final conclusions. Given the public availability of the ASES membership directory, this study focused only on active ASES members, excluding nonmember shoulder surgeons who may be more representative of community shoulder and elbow surgeons that rely more on patient ratings to recruit new patients. Likewise, the selective criteria for active membership may result in biased reporting. Selective inclusion criteria in our study may also underrepresent social media usage for platforms like YouTube, where institutional accounts were excluded. There were no additional controls for potential confounding variables such as patient demographics, clinical outcomes, or quality of patient reviews. In addition, the patients who are leaving reviews may not be the same individuals that are likely to find their shoulder surgeon on social media.^13^ We must consider that patients may be hesitant to engage with their surgeons on social media due to privacy concerns, so higher social media engagement for surgeons may be due to engagement by one's friends, family, or colleagues rather than patients. Higher ratings for younger surgeons may also be due to a younger patient demographic that is likely to leave reviews and engage with their physicians online. Inherent biases remain in patient reviews, as there is a tendency for individuals to write either heavily favorable or unfavorable ratings and comments on PRWs, meaning they may not truly reflect a patient's experience or a surgeon's expertise. Future studies that are looking to expand upon our results should also look at understanding the correlation between patient satisfaction and surgical outcomes.^21^

## Conclusion

Patient satisfaction for shoulder surgeons plays an important role in patient engagement, retention, and recruitment, as well as in surgeon and hospital reputation. Nonsurgical factors such as experience level, practice setting, social media utilization, and research productivity may also play a positive role in patient ratings and engagement on PRWs. Additional factors such as training prestige, region of practice, and fellowship type are not likely to influence patient ratings. Shoulder surgeons can consider these factors as complements to their clinical practice to optimize patient satisfaction and online engagement.

## Disclaimers:

Funding: No funding was disclosed by the authors.

Conflicts of interest: Michael B. Gottschalk receives institutional support from Skeletal Dynamics, Acumed, and Arthrex; He has received research support from Stryker and Konica Minolta; is a board or committee member of the American Society for Surgery of the Hand; and is an editor for the Journal of Hand Surgery and Surgical Techniques in Orthopedics. Eric R. Wagner receives consulting fees from Stryker, Biomet, Acumed, and Osteoremedies and receives research support from Arthrex and Konica Minolta. The other authors, their immediate families, and any research foundations with which they are affiliated have not received any financial payments or other benefits from any commercial entity related to the subject of this article.
